# Adaptive flood risk management planning based on a comprehensive flood risk conceptualisation

**DOI:** 10.1007/s11027-015-9638-z

**Published:** 2015-03-12

**Authors:** Frans Klijn, Heidi Kreibich, Hans de Moel, Edmund Penning-Rowsell

**Affiliations:** 1 Deltares, PO Box 177, 2600 MH Delft, The Netherlands; 2 GFZ German Research Centre for Geosciences, Potsdam, Germany; 3 Amsterdam Free University, Amsterdam, The Netherlands; 4 Flood Hazard Research Centre, Middlesex University, London/Oxford University, London, England USA

**Keywords:** Adaptive delta management, Delta programme, Exposure, Flood risk, Future scenarios, the Netherlands, Robustness, Spatial planning, Tipping points, Vulnerability

## Abstract

Densely populated deltas are so vulnerable to sea level rise and climate change that they cannot wait for global mitigation to become effective. The Netherlands therefore puts huge efforts in adaptation research and planning for the future, for example through the national research programme Knowledge for Climate and the Delta Programme for the Twenty-first century. Flood risk is one of the key issues addressed in both programmes. Adaptive management planning should rely on a sound *ex-ante* policy analysis which encompasses a future outlook, establishing whether a policy transition is required, an assessment of alternative flood risk management strategies, and their planning in anticipation without running the risk of regret of doing too little too late or too much too early. This endeavour, addressed as adaptive delta management, calls for new approaches, especially because of uncertainties about long-term future developments. For flood risk management, it also entails reconsideration of the underlying principles and of the application of portfolios of technical measures versus spatial planning and other policy instruments. To this end, we first developed a conceptualisation of flood risk which reconciles the different approaches of flood defence management practice and spatial planning practice in order to bridge the gap between these previously detached fields. Secondly, we looked abroad in order to be better able to reflect critically on a possible Dutch bias which could have resulted from many centuries of experience of successful adaptation to increasing flood risk, but which may no longer be sustainable into the future. In this paper, we explain the multiple conceptualisation of flood risk and argue that explicitly distinguishing exposure determinants as a new concept may help to bridge the gap between engineers and spatial planners, wherefore we show how their different conceptualisations influence the framing of the adaptation challenge. Also, we identify what the Netherlands may learn from neighbouring countries with a different framing of the future flood risk challenge.

## Introduction

Accelerated climate change may affect societies in various ways, through higher temperatures, rising sea level, more frequent storms, more frequent river floods and higher flood levels, more prolonged droughts, etc. Especially, deltas are very susceptible to these effects, for various reasons, first and foremost because these low-lying coastal areas are threatened from various sides: from the rising sea, from the rivers which drain vast hinterlands and from above by more intense rainfall. If we extend the scope, however, from climate change only to a broader scope of geo-ecological changes, it appears that subsidence and disturbed sediment supply may be equally or even more important issues in deltas. For example in Jakarta, Indonesia, subsidence is in the order of 0.1 myr^−1^ (Brinkman and Hartman 2008), which is much faster than the sea level rise of 0.01 myr^−1^. Similar rates are found elsewhere (Erkens et al. 2014). This may cause the drowning and subsequent loss of land through increased coastal erosion, which is aggravated by the sediment depletion resulting from trapping behind dams and the regulation of rivers (cf. Syvitski et al. 2009), for example in the Mississippi delta where the little remaining sediment also disappears into the gulf as the main discharge is channelised via the birdfoot.

If we shift from the geo-ecological subsystem to the socio-economic subsystem of deltas, it appears that deltas also attract many people and show rapid economic development because of their fertile soils, easy access to markets via waterways and sea ports, and ease of construction on relatively flat surface. This explains the above-average development rate of deltas, coastal fringes and river floodplains (Jongman et al. 2012), and it is also the main explanation of increasing economic losses as a result of floods (FLOODsite 2009a).

The vulnerability of deltas to climate change implies that these areas cannot wait whether a global mitigation policy has any effect, but that they need to adapt in order to survive. This certainly applies to the Netherlands, which can be considered as one big delta of the rivers Rhine, Meuse and Scheldt, but especially as over 55 % of the country’s surface area is flood-prone, whereas 40 % lies below mean sea level. It explains why the Netherlands pays due attention to climate change for several decades already. In the national programme Adaptation of Spatial Planning to Climate Change (ARK: Adaptatie Ruimte en Klimaat), it was established that the largest challenges from climate change relate to flood risk management and freshwater (resources) management (Kwadijk et al. 2006).

This made the Netherlands’ government solicit advice from an independent committee, the so-called Second Delta Committee. This committee advised on a Delta Programme, a Delta Fund and a Delta Commissioner to be installed, in order to ensure that a long-term adaptation strategy be drafted for integrated water management and spatial planning in view of a definitely changing climate, a surely rising sea level and probably changing river discharge regimes (Delta Committee 2008). These advices were followed up, which means that the Delta Programme—which formally started in 2010—can be considered as the national authorities’ response to the Second Delta Committee’s advice. It addresses the key question of how to ensure a sustainable flood risk management and freshwater resources management for the remainder of the century. The leading thought is that the country should anticipate instead of reacting on a disaster afterwards—compare Hurricane Katrina causing US$100–125 billion of direct damage or Hurricane Sandy causing 65 billion US$ damage (NatCatSERVICE, Munich RE 2013)—as this is economically preferable. The Delta Programme will propose the first key policy decisions in 2014.

The Delta Programme is being scientifically supported not only by dedicated studies but also by the research programme knowledge for climate change (KfC), which runs in parallel. This addresses the majority of climate-change-related adaptation challenges, on both floods and droughts, for urban and countryside environments and functions, and covering various scientific fields from climatology to decision support and governance. The character of the research is more strategic, in contrast to the applied research which directly supports the Delta Programme. This special issue is dedicated to the results of theme 1—on flood risk management—of this KfC research programme.

Obviously, neither the Netherlands’ policy making nor our research activities have developed in isolation. Here, we only mention some recent relevant developments, such as, firstly, the European Union (EU) framework directive on Flood Risk Assessment and Management, issued in 2007 (Directive 2007/60/EC), which requires all member states to perform preliminary flood risk assessments by 2011, to map flood hazard and flood risk by 2013, and to draft flood risk management plans by 2015. This activity has been supported by, secondly, the development of scientific concepts, methods and tools via the largest EU Integrated Project on flood risk ever, FLOODsite (Samuels et al. 2010; FLOODsite 2009a), which significantly influenced the way of thinking and scientific approaches in the field of comprehensive flood risk management (Klijn and Schweckendiek 2013).

Finally, an EU Adaptation Strategy has been issued in 2013, which promotes member states to adopt comprehensive adaptation strategies, aims to ensure that Europe’s infrastructure is made more resilient and enhances knowledge exchange through Climate-ADAPT (European Climate Adaptation Platform). Obviously, the Netherlands’ Delta Programme may qualify as the Dutch comprehensive adaptation strategy, especially if it would succeed in mainstreaming climate adaptation into flood risk management, freshwater resource management and spatial planning.

A first challenge then is to bring together different perceptions of what constitutes flood risk, how it develops, what the causes of this development are, how they can be influenced to reduce the risks—by which points of attack and by which measures—and how these should be combined. This entails the framing of the problem and its causes and the framing of the portfolio of measures and strategic policy alternatives. In this paper, we propose a scheme which reconciles different concepts of flood risk, and we show how this may influence the framing of the adaptation challenge. In subsequent sections, we show how it influences the view on risk development and the selection of measures. And, we show how the other papers in this special issue relate to it. Before proposing this conceptual scheme in the core of this paper, we first give a brief overview of recent developments in tackling the adaptation challenge, and we recall the key concepts of flood risk analysis and principles of its management.

## The adaptation challenge and how this is tackled

Drafting an adaptation policy requires thorough insight in the precise kind and magnitude of the future problems, the possibilities to counteract or reduce these in terms of strategic alternatives composed of physical measures and policy instruments, and an assessment of how they perform, against what costs and with which unintended side effects and opportunities. This sequel of activities is often addressed as a policy analysis, in the sense of an analysis in behalf of planning and policymaking (after Thissen 1997; Walker 1986, 2000). How it can be applied for flood risk management planning has been shown by Klijn et al. (2012b).

The future problems in terms of increasing flood risks are usually estimated by applying a scenario approach (Evans et al. 2004a, b; FLOODsite 2009a; Klijn et al. 2012a). The Intergovernmental Panel on Climate Change (IPCC) scenarios for emissions, greenhouse gas concentrations, sea level rise and hydrological consequences such as changes in rainfall, evapotranspiration and river discharge hydrographs are well known and key for estimating possible flood frequencies and flood levels (see IPCC 2014). The further down the chain of effects, however, the larger the uncertainties about the degree of an effect (Haasnoot 2013) and sometimes even about the direction. Moreover, when climate changes beyond the range of the historically observed, unforeseen shifts may occur which complicate the downscaling of global climate scenarios to the national or regional level: Uncertainty increases to such a degree that the Netherlands’ Royal Meteorological Institute (KNMI 2006, 2014) refuses to specify the probabilities of their four scenarios.

Even more uncertain are the autonomous developments in the socio-economic subsystem. For demographic, economic and land-use developments, a scenario approach may also be applied. For the Netherlands, a similar set of four scenarios has been developed (cf. Klijn et al. 2012a), but these are even more uncertain because of the global scale of economic developments and the dependency on the very dynamic geo-political changes. Specifically for the Netherlands’ Delta Programme, scenarios for geo-ecological change (climate change, sea level and subsidence) and socio-economic change (demography and economy) have been combined into so-called delta scenarios for 2015 and 2100 (Bruggeman et al. 2013), as a scenario approach at least allows a thorough exploration of possible futures (Haasnoot and Middelkoop 2012): in which scenario do we have a problem, about when, and about how big?

So far, this is all mainstream approach, which brings us to the question: What is new? We feel that the main innovations lie in what is called Adaptive Delta Management (Delta Programme 2011), which, of course, comprises adaptive flood risk management. This differs from the many centuries of adaptation in the past and from what is usually described in literature as the essence of adaptive management (e.g. Holling 1978; McLain and Lee 1996; Pahl-Wöstl et al. 2007; Medema et al. 2008) in that it (1) is anticipatory instead of responsive and (2) explicitly recognises uncertainties about the future and takes these into account in the management planning. Where conventional adaptive management may rely on trial-and-error and sound monitoring of developments, adaptive delta management is faced with the fact that, for example in the design flood levels, the signal of climate change cannot be derived from measurements soon enough against the large noise of natural climate variability (Diermanse et al. 2010). This calls for *ex-ante* assessments and anticipatory planning. Obviously, concepts such as decision robustness (Kwadijk et al. 2006; Lempert and Collins 2007; De Bruijn et al. 2008; Mens et al. 2011; Haasnoot 2013) and flexibility (Kwadijk et al. 2006; Van Rhee 2012; DiFrancesco 2013; Woodward et al. 2014) are key in this approach.

Van Rhee (2012) mentions four principles for adaptive delta management, which we very freely translate, namely the following:

- Short-term decisions should contribute to long-term objectives.
- Search for adaptation pathways with successive decision points in time rather than aim for a final situation at some point in the future (‘blue-print planning’) to allow for adaptation over time.
- Seek and value flexibility in individual measures and comprehensive strategies in order to allow for speeding up or slowing down and to prevent regret of underperformance or overinvestment and related to this.
- Aim for synergies with goals and development initiatives by other public and private parties, which reduces the likelihood of regret because of the other benefits achieved.

Although these principles may sound very obvious, especially the first one, their practical application is not without complications. We therefore refer to two interesting new concepts which were developed in support: the policy tipping points (Kwadijk et al. 2010) and the adaptation pathways (Haasnoot et al. 2013; Haasnoot 2013).

The policy tipping points approach involves exploring at which degree of climate change (or any relevant climate parameter) the current policy (or individual measures or constructions) is no longer suitable or acceptable and needs to be replaced by an alternative strategy or measure. By thus identifying the physical threshold levels which require a policy transition, one becomes less sensitive to the constant updates of climate and socio-economic scenarios every few years. It suffices to compare the threshold level with the new prognoses to identify the moment in future that a policy is no longer satisfactory (cf. Ranger et al. 2010).

The adaptation pathways approach (Haasnoot 2013) can be understood as a further development of the decision pipelines proposed in the context of the Thames 2100 study (ref; cf also Haigh and Fisher 2010). The approach involves mapping all the policy options (measures) and identifying until not only which degree of change they perform adequately (cf. the tipping point approach) but also whether a change of strategy or a change towards other measures is possible and when it should take place at the earliest or latest. It thus helps to identify possible lock-ins and elements of flexibility.

Both the policy tipping point approach and the adaptation pathways are being applied in the Netherlands’ Delta Programme, specifically for flood risk management and freshwater resources management. It was found (Passchier et al. 2009; Klijn et al. 2012a) that the current flood risk management policy, which relies almost entirely on flood protection through embankments and coastal sand nourishment, can be sustained for several centuries at least. However, as Klijn et al. (2012a) argue, there may be other reasons for a policy transition, namely that alternative management policies perform better, at lower cost and with more other benefits. This entails implementing other combinations of measures than applied in the past, including measures never applied before in the Netherlands (or very long ago), as well as improvements to well-known conventional measures.

This is what the KfC research programme on flood risk management intended to focus on, as evidenced by the various other papers in this issue. In this paper, we now return to the key concept of flood risk, because the way this is framed largely determines the portfolio of measures which is seriously taken into consideration.

## Reconciling different flood risk concepts

Before we can adequately frame the challenge of adaptative flood risk management, we need to agree on what we mean by flood risk and by flood risk management. This may seem a simple matter of looking into the relevant literature, but this will reveal that many different definitions exist and especially that very different notions exist. Actually, flood risk and its constituents prove to be ambiguous concepts.

As FLOODsite put substantial effort in defining both flood risk and flood risk management, we start from its Language of Risk. Based on numerous definitions in recent literature, FLOODsite (2009b) recommends to define flood risk as (freely quoted)

$$ \mathrm{risk}=\mathrm{probability}\left(\mathrm{of}\ \mathrm{flooding}\right)\times \mathrm{consequences}\left(\mathrm{of}\ \mathrm{flooding}\right) $$

This definition is preferred among natural scientists and especially among engineers, who usually strive for a reduction of the probability of flooding by means of flood protection and hence need to be able to calculate risks.

An alternative definition, which can also be traced back to a wealth of recent literature (cf. FLOODsite 2009b), is

$$ \mathrm{risk}=\left(\mathrm{flood}\right)\mathrm{hazard}\times \mathrm{vulnerability}\left(\mathrm{of}\ \mathrm{the}\ \mathrm{society}/\mathrm{area}\right) $$

**Fig. 1:**
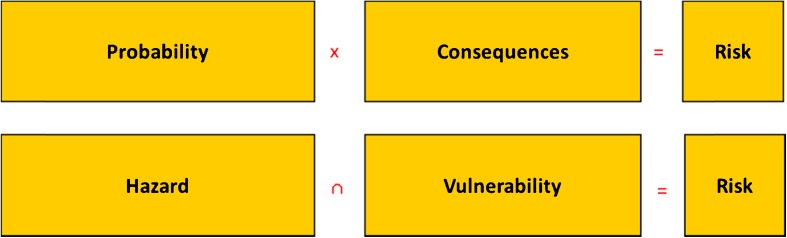
Flood risk can be conceptualised as a multiplication of flood probability and consequence or as geographic overlay of hazard and vulnerability

This definition is often preferred by social scientists and especially among planners, who usually regard the hazard as a given and spatial planning and influencing people’s behaviour as the means to adapt to that given. Both definitions are given in Fig. 1.

**Fig. 2:**
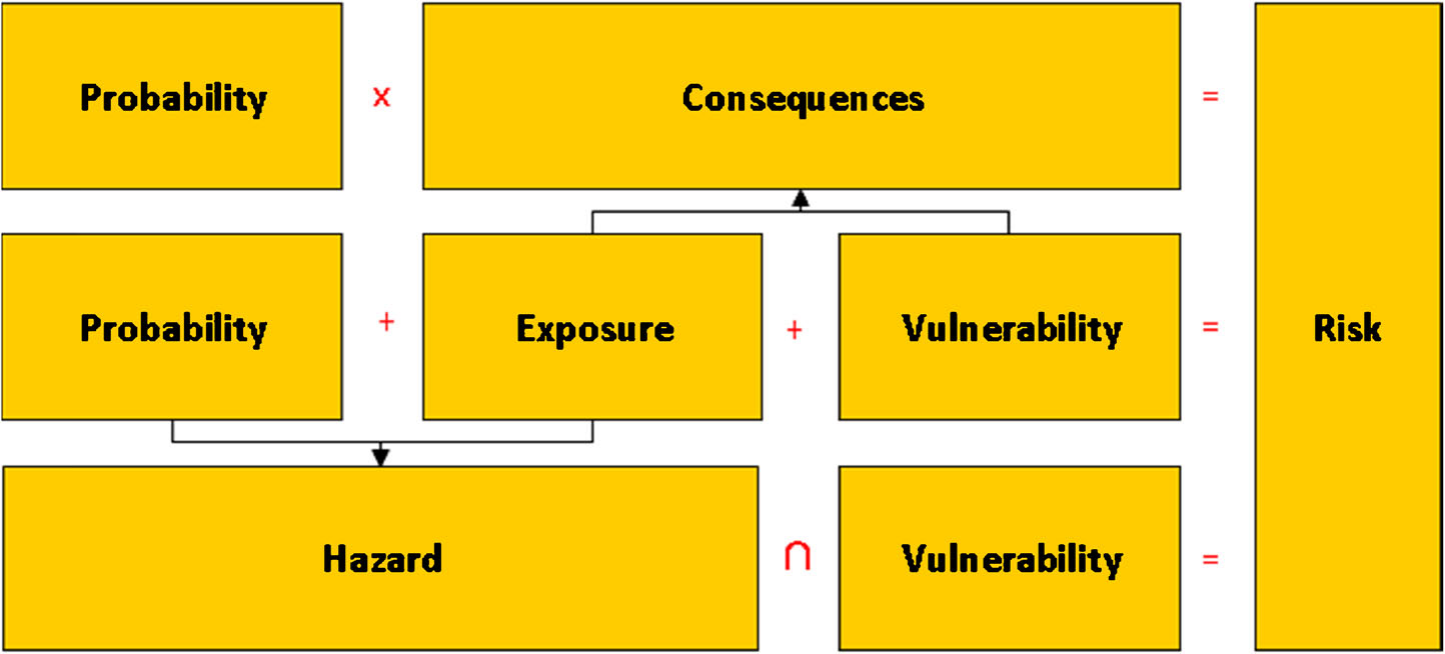
Explicit recognition of the flood’s characteristics under the term exposure allows reconciling the alternative definitions. Risk then becomes a combination of three key constituents: flooding probability, exposure determinants and vulnerability of receptors

The first definition (probability × consequence) has long been the only accepted definition among the engineers of Rijkswaterstaat and in the former Ministry of Public Works and Water Management of the Netherlands. It very much suits the Netherlands’ situation, where about 3000 km of primary flood defences protect the majority of the flood-prone area. By multiplying the probability of a defence breach with its consequences, a quantitative estimate of flood risk can be obtained, where the vulnerability of the area and the flood’s extent and depth are combined into one figure for consequence (or some figures to distinguish between economic damage, number of fatalities and other risk metrics). Therefore, in Figs. 1 and 2, we chose the multiplication symbol as indicative operator.

In the second definition, the flood’s possible extent and depth (and other flood characteristics including probability) are all covered by the word hazard. FLOODsite defines a natural hazard as a natural phenomenon with the potential to harm. Actual harm can only occur to a vulnerable society or area. In the EU Floods Directive, hazard mapping is given a prominent place, alongside with risk mapping by means of overlay. Therefore, the indicative operator we chose in Figs. 1 and 2 is the overlay symbol.

The key difference between the two definitions thus resides in where the characteristics of the flooding are being incorporated. They are obviously hazard characteristics in the second definition, but in the first definition, the characteristics of the flooding are largely captured within the term consequences. We suggest that explicitly introducing the element of exposure may perhaps help here. and indeed, at a closer look, it turns out that FLOODsite (2009b) refers to many definitions of risk which contain the term exposure, sometimes between brackets as a sign of doubt about its precise character or as to its place. For the term exposure, FLOODsite (2009b) refers to one earlier definition only and (on p. 15) recommends ‘quantification of the receptors that may be influenced by a flood (for example, number of people and their demographics, number and type of properties etc.)’. According to this definition, exposure is rather the result of overlaying a flood’s (or all possible floods’) footprint(s) on a map of receptors than a constituent of risk by itself. Thus, it is more or less identical to elements at risk, another frequently used term in many definitions of risk (cf. FLOODsite 2009b). This means that exposure is determined by the presence of receptors (cf. also UNISDR 2009) as well as their character—e.g. their vulnerability—on the one hand, and characteristics of the flooding—which we shall call exposure determinants (or abbreviated, exposure) in this paper—on the other. Thus, we may gain a broader understanding of exposure than simply a number of potentially affected receptors. After all, there is a substantial difference between a wet kitchen floor and being submerged.

In the first definition, exposure determinants, such as water depth and extent, are included in the term consequence, because they are indeed a hydraulic consequence of a breach and hence required for the calculation of the consequences of a breach in terms of economic damage or number of fatalities. In the second definition, flood depth and extent are hazard characteristics, alongside with probability. By explicitly distinguishing exposure determinants as a separate constituent of flood risk, the two competing definitions and schools can be reconciled (Fig. 2).

According to this scheme, consequences can be understood as comprising both exposure and vulnerability, whereas hazard comprises both probability and exposure determinants. Even then, each term may need a more precise definition, as the terms for the constituents remain ambiguous by themselves.

For example, probability may equally refer to the probability of an embankment breaching, to the probability that a certain location is being exposed to flood water, or finally to the probability of consequences. In the latter case, probability swallows the other concepts. We therefore prefer to delimit its use to the first definition, unless stated otherwise: thus, probability that flooding occurs (or breaching in the case of embankments), which is not the same as flood probability as this may merely mean high water in a river or at sea (FLOODsite 2009a).

Ambiguity is also attached to the term consequence. Of course, a breach in an embankment is a geo-technical consequence, even when the impacts on society are nil. But, in flood risk management, this is not a relevant consequence. We therefore prefer to follow FLOODsite’s (2009a) more encompassing definition, which requires relevant societal consequences, such as economic damage or loss-of-life, to result from a flooding before one could speak of flood risk, which then is, obviously, not equal to the risk of defence failure.

Vulnerability may also refer to various entities. It may refer to persons or property, such as houses or cattle, which are then equally vulnerable whether on a hilltop or in a floodplain, as the characteristics of the person or property are determinant. Others use the term vulnerability for an entire area while including the area’s characteristics such as elevation, flood defence infrastructure, etc. (e.g. Marchand 2010) and thus excluding all areas above maximum flood level. This results in the concept of exposure being swallowed by the definition of vulnerability. The opposite also occurs, e.g. by Jongman et al. (2012) who show how the vulnerability of flood-prone areas worldwide increases as a consequence of demographic and economic development, but they call it increasing exposure (following the definition of IPCC 2012 or FLOODsite 2009b).

We prefer to reserve the term vulnerability for the people, their property (e.g. buildings) and their activities in an area (areal vulnerability), as the distinction between hazard and areal vulnerability has certain obvious advantages for spatial planning (cf. Van de Pas et al. 2012; Pieterse et al. 2013) and disaster management planning (UNISDR 2009).

The proposed limited interpretation of the terms probability and vulnerability allows us to explicitly look into exposure determinants—or the characteristics of the flooding process and pattern—as a separate constituent of risk as well as to investigate measures to influence it. Exposure determinants of importance then comprise flow speed, water level rising rate, time span between breach and arrival at location, maximum depth, final depth and extent, and time span before drying out again. For the Netherlands, these hazard characteristics have recently all been mapped in the context of complying with the EU Floods Directive.

### How concept and framing relate

In a separate supplement to the National Water Plan, the Netherlands’ government adopted what it calls a multi-layered safety approach (*meerlaagsveiligheid*) or rather a multiple-tiered approach to flood risk management, as follows:

1. Flood protection^1^ as keystone
2. Sustainable spatial development as supplement
3. Disaster management to finish it off

Taking flood protection as keystone can be regarded as a continuation of a policy which was drafted already some 50 years ago and which is legally embedded in the Water Law (the successor of the Law on Flood Defence). The second layer aims to prevent a further rise of flood risks through demographic and economic developments in the future, whereas the third layer is meant to reduce the effects of any undesired flooding event.

The concept of risk which obviously lies behind this is the first definition (risk = probability × consequence), with probability (of failure of the defences) in layer 1, and consequences in terms of damage to property and economy, respectively people, in layers 2 and 3. This is reflected in the dominant studies initiated by the Delta Programme.

**Fig. 3:**
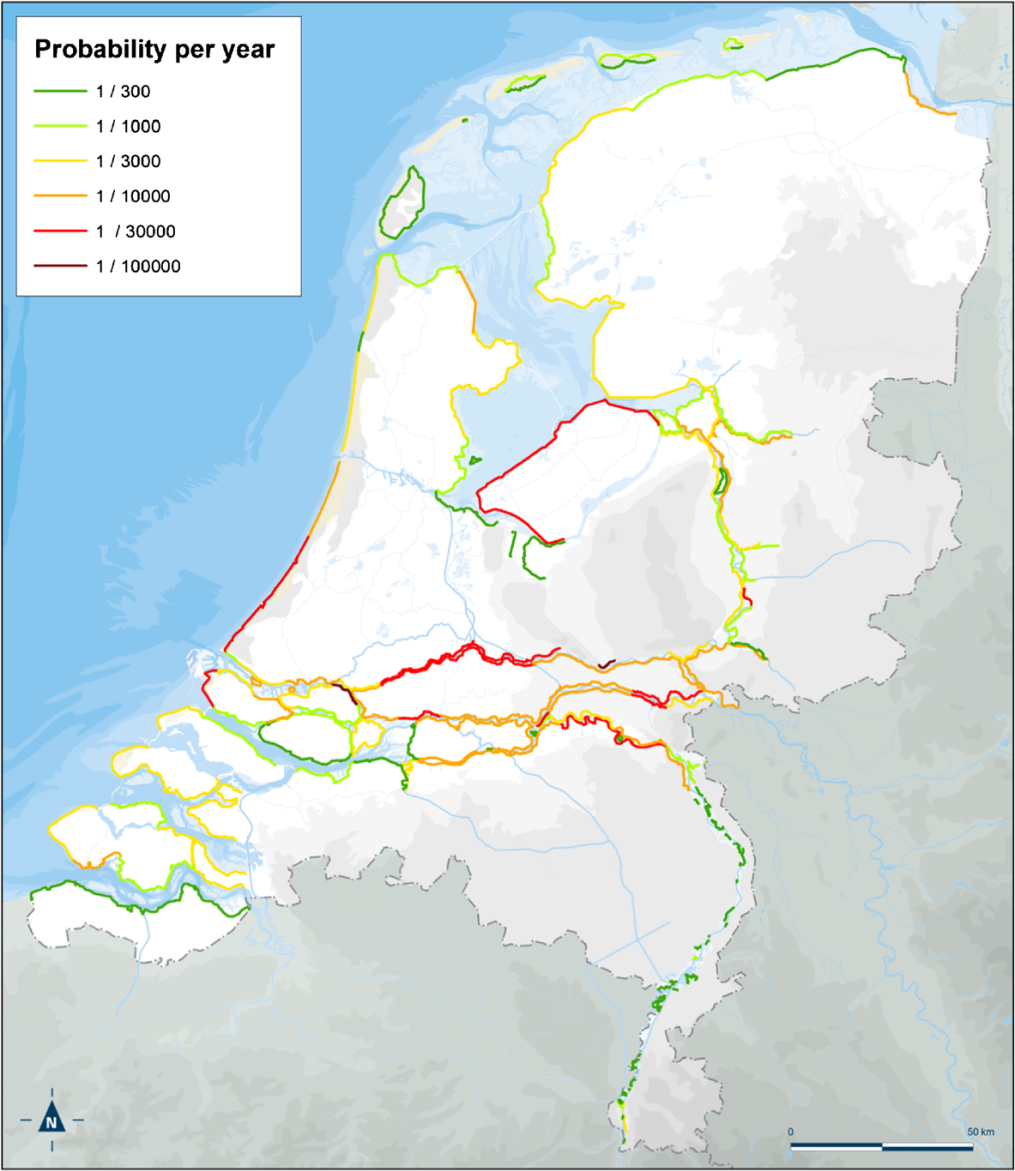
Proposal for new protection standards based on flood consequences as input for cost-benefit analysis (economic optimum, cf. Kind 2013)

The Delta Sub-Programme dedicated to flood risk management, for a start, had the flood consequences analysed in detail (De Bruijn and Van der Doef 2011; Beckers and De Bruijn 2011) and then put these central to derive appropriate levels of flood protection (Kind 2011). These protection levels, in terms of economically optimal flooding probabilities, were calculated by applying a cost-benefit approach, which aims at defining the protection level that yields the lowest residual risk against the lowest total costs of investment and maintenance (Kind 2013; Gauderis et al. 2013). This approach thus relies on consequences as a given and focuses on defining acceptable probabilities of flooding, i.e. measures for flood protection. Recently, this approach has been fine-tuned in search of the optimal spatial scale for which standards should be set, which has become possible with the progress of the FLORIS project (or VNK in Dutch): FLOodRISk in the Netherlands (Projectbureau VNK 2011). This project investigates in detail the probability of failure of flood defences taking into account all relevant failure mechanisms, the consequences of a large number of possible breaches and the combination of these in terms of risk: fatality risk and economic risk. This zooming in—or rather spatial differentiation—allows a better prioritisation of reinforcing flood defences in such a way as to obtain the best value for money. The result in terms of proposed protection levels^2^ is given in Fig. 3.

We may thus notice that in this approach, the socio-economic development of vulnerability is taken for granted, as well as the increased exposure due to higher flood levels at sea and in the rivers. The adaptation challenge is thus framed as a flood protection challenge: It is the water that is to blame, and it is the water that should be controlled.

**Fig. 4:**
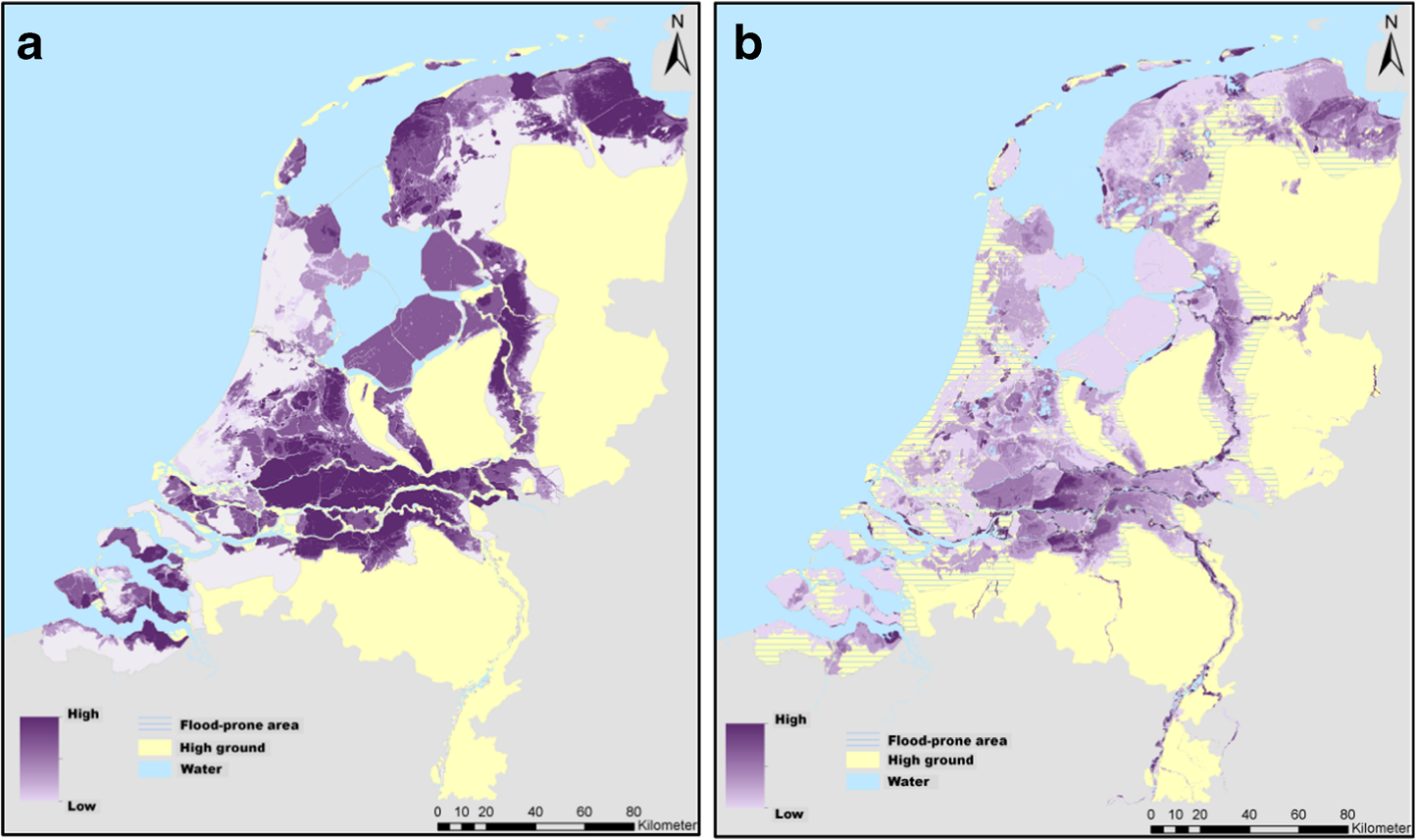
Local flood fatality hazard (**a**, *left*) based on modelling hypothetical loss-of-life (after Beckers and De Bruijn 2011) and local flood damage hazard (**b**, *right*), based on modelling hypothetical damage (after Van de Pas et al. 2012)

Another study we want to refer to in this context, was initiated by the Delta Sub-Programme on Urban Development and Re-development and executed jointly with our KfC-consortium. Van de Pas et al. (2012) focused on how to achieve a useful and sufficiently detailed map of flood hazard in support of spatial planning: i.e. measures that reduce the vulnerability of flood-prone land. They therefore started from the other side by taking hazard as a given for spatial planning purposes. This mapping exercise entailed combining information on the probability of flooding and on exposure characteristics, in order to achieve meaningful hazard maps. This was the second attempt to systematically map flood hazard—in all its dimensions—for the whole country, after a first approximation by De Bruijn (2007; cf. also De Bruijn and Klijn 2009) and taking into account experiences elsewhere in Europe as inspiration (EXCIMAP 2007; De Moel et al. 2009). It resulted in many maps of exposure characteristics for various flood probabilities, as well as in two comprehensive hazard maps: one unifying all relevant parameters for decisions related to settlement and evacuation (Local Flood Fatality Hazard) and one unifying the relevant parameters that determine damage to property (Local Flood Damage Hazard) (Fig. 4a, b; Van de Pas et al. 2012; Pieterse et al. 2013).

In this approach, the hazard is considered as a geo-ecological given, whereas it is assumed that settlement and development can be influenced by spatial (physical) planning. Actually, this approach and conceptualisation of risk seem to be dominant in most other countries as evidenced by the public availability of hazard maps (Büchele et al. 2006; EXCIMAP 2007) and the way media use to cover disasters: Often, the planners and people are blamed. The adaptation challenge is thus framed as a planners’ challenge or partly as a challenge for individual action (Kreibich et al. 2011a; Bubeck et al. 2012; Bubeck 2013).

In hindsight, we may thus conclude that within the Delta Programme, two contrasting risk conceptualisations and frames have been used by two sub-programmes. In both frames, exposure is neglected, however, as those who deal with flood protection consider it part of consequence reduction—and not their responsibility, whereas the spatial planners consider it part of the hazard—and hence not their responsibility either. We think this is unfortunate, as thus no party feels—and can be held—responsible to seriously consider the possibilities of influencing the breach development, the inflow rate and volume, the flood water’s pathway, etc.

In our KfC research we therefore adopted our richer conceptualisation of flood risk, with explicit acknowledgement of exposure. How this translates into the analysis of future flood risk and how it may widen the scope of potential risk reducing measures taken into account will be illustrated below.

### The development of flood risk constituents in the future

By successively focusing on changes in the probability of flooding, in the exposure characteristics of future floods, as well as in the vulnerability of the flood-prone area, it is possible to obtain a thorough understanding of future flood risk, both in quantitative terms and in terms of geographical distribution. Over the last decades, many investigations have been carried out in different parts of the world, the results of which have been collected in the context of the IPCC 5th Assessment report (IPCC 2014). Here, we shall merely illustrate the process by primarily referring to projects we have been involved in ourselves.

As for probability, Klijn et al. (2004, 2012a) established for the Netherlands an increase in hazard probabilities on the basis of scenarios for sea level rise and river discharge regime. It was found that, without counteracting measures, the exceedance probability of the design water level might increase with a factor of about 2–3 on average by 2050 along the rivers, the coast, in the estuaries, and on Lake IJssel. Another doubling or tripling may be expected by 2100. This increase of hazard probabilities, however, does not translate into an increase of flooding probabilities, as the current Netherlands’ policy requires that legal protection standards are met. This means that the actual flooding probability—or rather dike failure probability—is to be maintained through measures already foreseen or improvements to the defences yet to be designed (Klijn et al. 2012a).

For the UK, the changing probability of flooding was the central issue in both the Foresight Future Flooding study (Evans et al. 2004a) and in the analysis for the Thames Estuary (TE2100), which applied a similar approach (Environment Agency 2010). In the former case, changes in the flood probability of each 10 × 10-km grid square across the whole country were assessed and mapped, whereas in the latter case, rising sea levels were translated into flood probabilities in the estuary into the future, triggering increased overtopping of existing defences.

For Germany, investigations into an increase of flood probability were done on both past and future changes. Petrow and Merz (2009) detected changes in maximum flood flows between 1951 and 2002 which were most likely climate-driven, and at least consistent with changes in atmospheric circulation patterns (Petrow et al. 2009). They could, however, not detect a ubiquitous increase of flood frequency or magnitude. Into the future, an ensemble study in three small- to medium-sized river catchments in Germany also revealed a high uncertainty range of the change signal for the period 2021–2050 (Ott et al. 2013): For the Ruhr catchment (west), winter and summer discharges might increase, for the Mulde catchment (east), no future change in discharge regime was found, whilst for the Ammer catchment (south), it was found that winter discharges might increase, whereas summer discharges might decrease (Ott et al. 2013). Consequently, no flood probability prognoses for the totality of Germany are available yet.

**Fig. 5:**
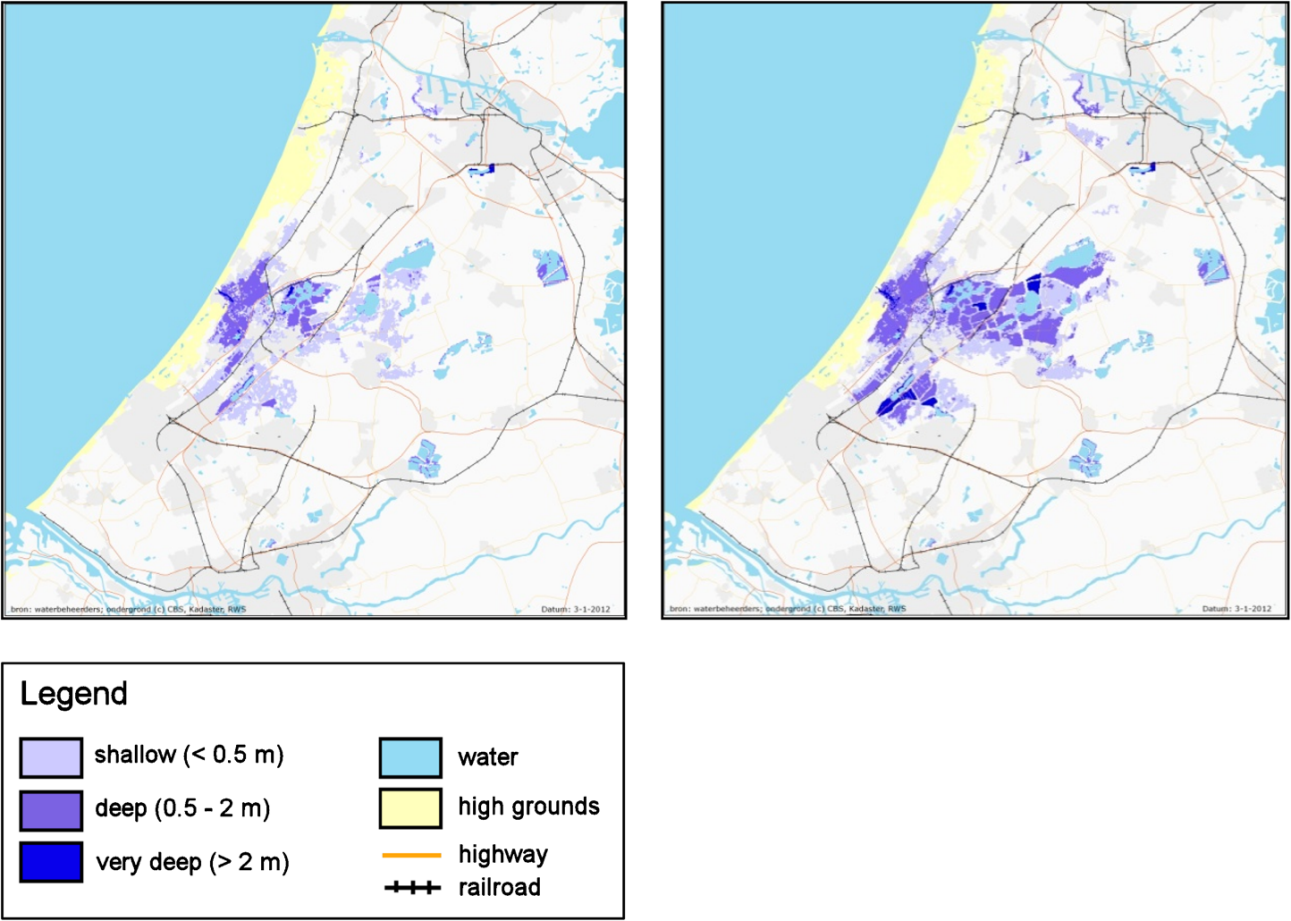
Maximum extent and maximum water depth after a breach at Katwijk for design storm surge level and waves in the present situation (*left*) respectively with a 85 cm higher mean sea level as expected by 2100 (*right*) (courtesy K.M. de Bruijn)

As for changes in exposure determinants, this was first neglected in the Netherlands but later assessed separately, because the fact that flooding probability did not increase called for a closer investigation of other consequences of sea level rise and increasing discharges. De Bruijn (cf. Klijn et al. 2010a; 2012a) performed a number of flooding simulations by which it was possible to establish the increase of both flooding depth and flood extent in a number of representative polder areas (Fig. 5) and to extrapolate to the remainder of the country. By subsequently calculating the resulting economic damage and number of fatalities for the present land use and population, we could establish the separate contribution of greater exposure to the possible future development of risk. It was thus found that increased flood extent and depth alone might cause the economic flood risk to rise by a factor of 1.7 at the maximum for some coastal areas by 2050 already (Klijn et al. 2010a, 2012a).

For the UK, changes in exposure determinants were not addressed explicitly, neither as part of the central estimates of risk in the Foresight Future Flooding project (Evans et al. 2004a) nor in the TE2100 study (Environment Agency 2010). As the majority of the UK flood-prone land consists of natural valleys without any flood protection, however, the Foresight project implicitly did include characteristics of the flooding, such as inundation depth and an increase in the size of the areas affected by future flooding, in its estimates of risk. Exposure increases were thus assessed as a larger number of properties that could be flooded with more severe events. This matches FLOODsite’s (2009b) definition of exposure as quantification of the receptors that may be influenced.

Also for Germany, it applies that changes in exposure as a consequence of changes in flooding pattern and process have not been investigated separately but only as integral part of increasing flood hazard due to climate change. Te Linde et al. (2011), for example, established the increase in flood risk as a consequence of a climate change scenario for the entire Rhine River but did not distinguish between the influence of increasing probability and increasing depth and extent of the flooding. Now, the Rhine River partly flows through an unprotected river valley, which makes such a distinction unnecessary, but other—very large—parts are protected by embankments. This might call for a separate analysis of probability and exposure determinants, as in the Netherlands.

As for vulnerability, Klijn et al. (2007, 2012a) established that for the Netherlands, this has been by far the most important cause of increasing flood risks in the past and may be the most important cause into the future as well, but of course depending on socio-economic scenario. Based on land use prognoses for 2040, which Netherlands Environmental Assessment Agency (Koomen and Van der Hoeven 2008) derived from four possible scenarios ranging from a shrinkage of population from the present 16.0 million to 15.8 million inhabitants (Regional Communities (RC)) to a growth to 19.7 million (Global Economy (GE)) and with a yearly economic GBP growth ranging from 1.2 % per head (RC) to 2.1 % per head (GE), we established the influence on the development of flood consequences. In the upper scenario, the economic flood risk was found to increase with a factor 2.3 by 2050 as a consequence of demographic and economic growth alone. For 2100, this could be translated into an increase with factor of about 8–10 in the high growth scenario (Klijn et al. 2012a). Similar findings have been reported from a number of local and regional analyses with comparable results (Maaskant et al. 2009; Botzen et al. 2010; Bouwer et al. 2010; De Moel et al. 2011), and recently Jongman et al. (2012) published a future outlook on the increase in vulnerability of all flood-prone areas worldwide. It was already established that the observed trend in disaster losses is primarily caused by the increasing vulnerability of floodprone areas due to their demographic and economic development (Bouwer 2011), and also, IPCC (2014) now reports that increasing vulnerability (in terms of exposed assets) is the main explanation for increasing flood risks.

For the UK, increasing vulnerability into the future was not assessed in the Foresight project or the TE2100 case study. The land use of the affected areas was assumed to remain constant, and assets at risk were not up-rated in value (or their susceptibility altered) with increasing future GDP levels. In the TE2100 analysis, socio-economic scenarios in the future were developed but more in the form of a sensitivity analysis than as part of the main assessment. The reason for the assumption of unchanging land use was the Government guidance for UK flood risk management appraisal that such changes should not be included, on the basis that flood risk management expenditure should not be seen to subsidise land development which would be the case if such development was to be counted towards scheme benefits (Penning-Rowsell et al. 2013).

In Germany, only few investigations were performed into changes of vulnerability to floods—all case studies. These comprise studies into land use change, which affects the vulnerability of the flood-prone area, as well as studies into changes in the vulnerability of individual objects as such. As an example of the first, Elmer et al. (2012) found that land-use change in the form of urban sprawl is the main driver of increasing flood risk in the lower part of the Mulde River Basin. It was found to cause an increase of the expected annual damage for residential buildings of 21 % between 2000 and 2020 for the maximum land-use scenario. Te Linde et al. (2011) calculated an increase of the entire Rhine basin’s flood risk as a consequence of increasing vulnerability of about 7–27 % until 2030 due to land-use change alone, depending on the socio-economic scenario. With respect to changing vulnerability of individual objects, research in Germany primarily focused on the response of households to reduce their vulnerability by taking precautionary measures after having experienced a flood (e.g. Kreibich et al. 2011b; Bubeck et al. 2012). This kind of research obviously does not address the question of autonomous changes in vulnerability to floods.

### Reducing flood risk and the different role of technical measures and policy instruments

**Fig. 6:**
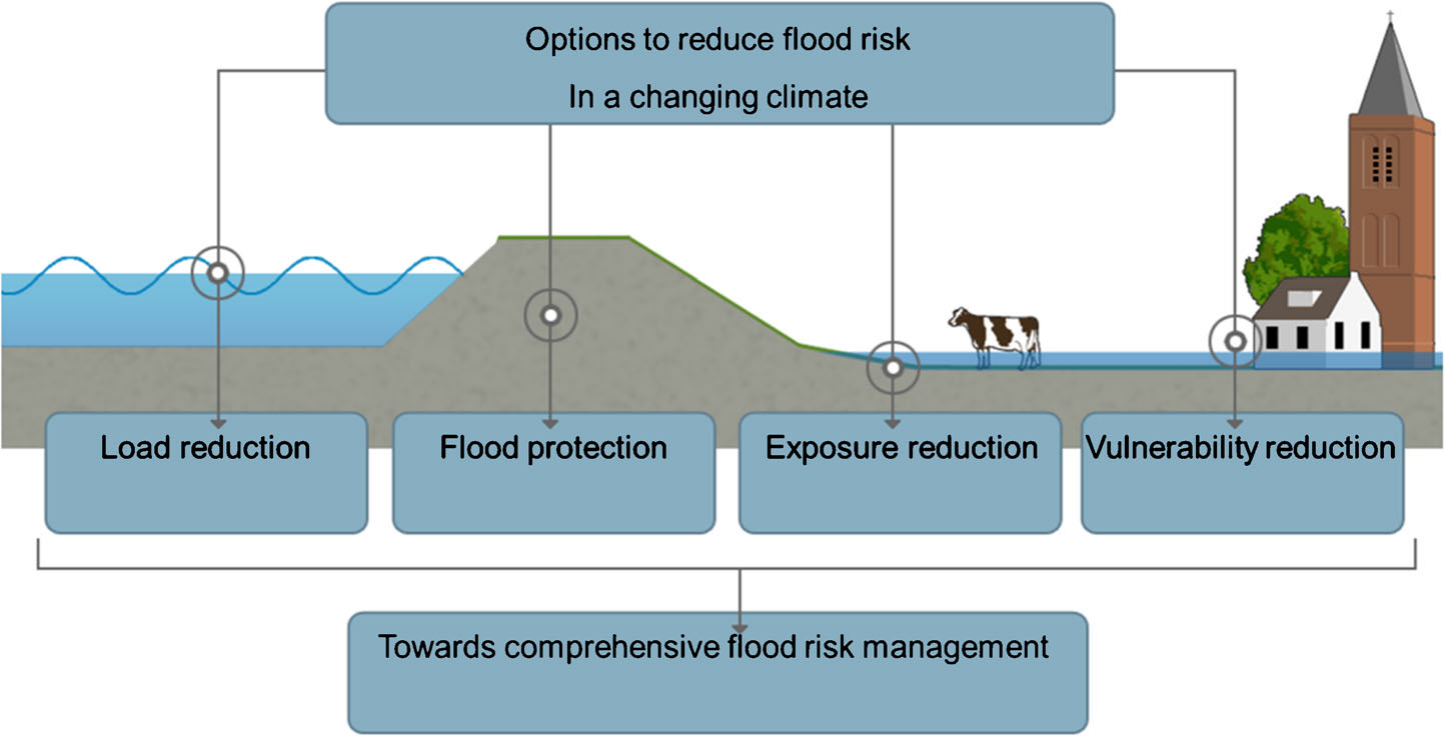
Measures and policy instruments aimed at reducing flood risk may reduce flood probability, the flooding process and pattern or the vulnerability of the protected land

A thorough understanding of how flood risk changes over time and insight in the relative contribution of the three recognised flood risk constituents may help to identify and select measures and policy instruments aimed at influencing the development of each of these. Before they can be combined into strategic alternatives for flood risk management policy, we also need unambiguous policy goals, as well as knowledge about the effectiveness, costs and side-effects of the possible measures and instruments that may reduce risk. The scheme of Fig. 2 may be used as an aid to categorise measures as reducing the probability of flooding, or its rate, depth, extension etc., or the vulnerability of the area affected. As the Netherlands has a tradition of flood defence, for which a distinction is made between influencing the load to the flood defences from their strength, we also made this additional distinction as depicted in Fig. 6.

Within our KfC research programme, we focused on relatively new measures, or measures which have received little attention in the Netherlands but are frequently applied elsewhere. Our main aim was to explore their cost-effectiveness and important side-effects or co-benefits apart from reducing flood risk. In the subsequent papers in this special issue, some of these measures are being discussed in detail.

First, however, De Moel et al. (2015) review various approaches to flood risk assessment, which is a prerequisite for any measure aimed at reducing risk. It entails assessments of—at least—economic risk and fatality risk.

Keijsers et al. (2015) focus on means to reduce the wave load on flood defences by means of beach nourishment—in front of natural dune coasts which provide flood defence—whereas Van Loon-Steensma (2015) investigate the possibility to enhance the development of vegetated forelands in saline coastal and fresh estuarine environments—in front of conventional embankments. Both measures not only reduce the wave load on the flood defences but also contribute to the strength of the defences as such by increasing the volume of the dunes and by increasing the macro stability of the embankments. They are often addressed as examples of building with nature (De Vriend and Van Koningsveld 2012).

The defences themselves are put central in the investigations of Nillesen and Kok (2015) and Tsimopoulou et al. (2015). The latter authors seek to optimise risk reduction and costs from an engineering point of view, which is still mainstream in the Netherlands, as it does not require any fundamental change of policy away from flood protection and towards a more comprehensive strategy. Within this same context, Nillesen et al. focus on the challenge of how to design embankments which protect against rare floods, while at the same time being sufficiently attractive elements for those who have to negotiate the embankments in their everyday life. With more than 3000 km of primary defences, embankments are a key element from an amenity point of view, often determining the scenic quality of the Dutch landscape. This calls for due attention for the possible impacts of reinforcements and requires a design approach.

Kreibich et al. (2015), in contrast, go into the possibilities of measures that aim at vulnerability reduction, more precisely damage reduction. Such measures are widely applied in Germany, with its many unprotected river valleys. According to section 5 of the German Federal Water Resource Act (Wasserhaushaltsgesetz 2009), every person that could be affected by a flood is obliged to undertake appropriate actions that are reasonable and within one’s means to reduce flood impacts. In the UK, development planning is being regulated through the National Planning Policy Framework (NPPF), which ‘sets strict tests to protect people and property from flooding which all local planning authorities are expected to follow. Where these tests are not met, national policy is clear that new development should not be allowed’. For the Netherlands, policy to prevent or regulate development is quite new and only applied in unprotected floodplains (e.g. Poussin et al. 2012). Within the Delta Programme some hazard zoning and related spatial planning policy is now being considered (Pieterse et al. 2013).

Penning-Rowsell and Priest (2015) address the issue of how to share the burden of the risk that remains after having taken all kinds of risk-reducing measures. They not only go into the possibilities of insurance, the common approach in the Anglo-Saxon world, especially the USA and UK, but also give their opinion on the advantages and disadvantages of a common pool, viz. the treasury filled by taxes and managed by the national government, as in the Netherlands.

In the above-mentioned contributions, exposure reduction by trying to influence the flooding process and pattern seems to have been neglected again. However, measures dedicated to reducing the inflow velocities, flood depth and flood extent are being considered in the final paper by Klijn et al. (2015), which primarily focuses on the assessment of comprehensive flood risk management strategies. The strategies taken into account comprise making room for rivers, which lowers the flood levels in such a way that a smaller area is flooded less deep, as well as making embankments virtually unbreachable, which limits the inflow rate and volume because they do not collapse but are only being overtopped during a shorter period of time (De Bruijn et al. 2013; Knoop et al. 2013). Compartmentalisation, which reduces the area which is being flooded, is not being discussed in this issue but has been published about elsewhere quite recently (Asselman et al. 2008; Klijn et al. 2010a).

## Discussion

In this paper, we illustrated how different ways of conceptualising flood risk result in a different framing of what causes the risk and how it can/should be tackled: by flood defence or by spatial planning? For the Netherlands, it was established that centuries of good experience with flood defence have resulted in a conceptualisation of flood risk as consequence multiplied by flood probability, which results in a bias towards flood defence as the obvious answer, also to the future challenge of adapting to increasing flood risk as a consequence of global change and socio-economic development. This might be qualified as narrow-mindedness and/or preoccupancy, associated with the conventional engineer’s framing of what constitutes flood risk in the Delta Sub-Programme on Flood Risk Management.

A planners approach with hazard maps and vulnerability as key constituents appears to be much more common in other countries, such as Germany, the UK and France. In these countries, responsibility is for a large part transferred to local communities (development planning), individuals (Kreibich et al. 2015), banks (mortgages) and industries (insurance; Penning-Rowsell and Priest 2015). A different framing of what constitutes risk (‘without people, no risk’) implies attention for measures to be seriously taken into account other than flood defence only. This approach is adopted in the Netherlands’ Delta Sub-Programme on Urban Development and Re-development but gains ground only very gradually, and it is still uncertain whether it will result in any significant concrete policy act.

We may thus conclude that the conceptualisation of flood risk determines the framing of what the future’s adaptation challenge actually is and also results in a preference for certain measures and policy instruments. By recognising the flooding process and pattern as a separate risk constituent under the heading of exposure determinants, we may achieve a richer understanding and analysis of what constitutes flood risk and how it will develop into the future, as well as identify more measures aimed at reducing the inflow volume and speed, and the extent and depth of the flooding. This may help to reconsider the Netherlands’ approach to flood risk management in view of changing geo-ecological boundary conditions and socio-economic developments. It is likely that a more comprehensive set of measures and policy instruments is opted for, in order to achieve a more robust flood risk system (Mens et al. 2014), which is not only efficient from an economic perspective but also effective in preventing disasters with many fatalities because it not only reduces the probability of flooding but also its hydrological and societal consequences.

This calls for a broader view on the aims of flood risk management: not only a focus on immediate economic benefits and the repairing of thoughtless or badly informed planning in the past by better flood protection but also attention for how we should ensure a more sustainable future by really anticipating developments in the next decades and even centuries and act upon these insights. A reframing of what flood risk management is about and a rethinking of how to meet the challenges of global change by adaptive delta management may be a good start for this.

## Footnotes

1 The Dutch tend to call this prevention, which deviates from the terminology used in the EU Floods Directive, where prevention stands for risk prevention through spatial planning. It reflects that in the minds of the Dutch (policy makers) preventing floods is (still) the key issue, instead of preventing risk.

2 The final proposal of the Delta Programme deviates from this line of economic reasoning by first and foremost providing a basic protection level to all individuals in protected area (cf. Van der Most et al. 2014), which reflects complementary equitability reasoning.
